# Editorial: Insights in plant abiotic stress: 2021

**DOI:** 10.3389/fpls.2022.1085150

**Published:** 2022-12-06

**Authors:** Luisa M. Sandalio

**Affiliations:** Department of Biochemistry, Cell and Molecular Biology of Plants, Estación Experimental del Zaidín Consejo Superior de Investigaciones (CSIC), Granada, Spain

**Keywords:** antioxidants, drought, high light, salinity, temperature, priming, reactive oxygen species, reactive nitrogen species

Plants, as a source of food, fuel and oxygen, are the basis of life on Earth. Currently, over a billion people suffer from malnourishment, while a similar number lack basic micronutrients, such as Zn, Fe and Cu, in their diet, which has a major impact on human health, with future forecasts being even more pessimistic; thus global yields could decline by up to 30 percent by 2050 (Hober and Negra, 2020). Soil deterioration and impoverishment, mainly due to environmental pollution and climate change, as well as the massification of cities, contribute to this problem and constitute an important challenge for agriculture in the future. One of the consequences of climate change is an increase in CO_2_ emissions, temperatures, flooding, drought and soil salinization. Another effect of anthropogenic activity is the accumulation of heavy metals and other contaminants which greatly harm all types of organisms. These processes will lead to a reduction in plant production and product quality. Under these conditions, innovative agronomic practices and genetic crop improvements are required. Given this scenario, it is necessary to deepen our understanding of the mechanisms that regulate plant responses to stress conditions, as well as contaminants, in order to improve plant and food production under these unfavourable conditions through the development and selection of more stress-tolerant cultivars or species.

As sessile organisms, plants have to endure a wide variety of environmental stresses during their entire life cycle. To do this, they have developed complex responses to abiotic stresses, which involve multiple interactions between hormones and other signalling molecules. The physiological, metabolic and molecular responses of crops to combined abiotic stresses appear to be significantly different from individual stresses. The study of the mechanisms involved in plant acclimation to environmental stresses has become one of the most important areas of plant research. Metabolic imbalances induced by external stimuli, such as drought, high light intensity, salinity, flooding, high and low temperatures, promote the generation and accumulation of reactive oxygen species (ROS); this may lead to oxidative damage to proteins, lipids and nucleic acids, although these compounds also act as signal molecules at low concentrations (Mittler, 2017; Phua et al., 2021). The mechanisms available to the cell to perceive environmental and physiological changes are complex and not well known, which is one of the most challenging issues in plant biology.

Although the effects of single abiotic stresses on crops have been widely studied, combined rather than individual abiotic stresses usually occur in the field. Roots, the underground organs directly in contact with the soil, sense many of these abiotic stresses. In this area of research, Sánchez-Bermudez et al. (2022) have drawn up a review which summarizes current knowledge regarding the effects of combined abiotic stress in the root system of crops. The authors describe individual responses to specific abiotic stresses and how these responses are integrated when crops are challenged by combinations of different abiotic stresses. The authors have updated our knowledge of changes in the root system architecture (RSA) and physiology influencing both crop productivity and yields; they also provide the latest information on the key molecular, hormonal and genetic regulatory pathways that underlie root responses to these combinatorial stresses. An emerging approach to this issue is an understanding of plant stress memory and priming and their potential role in cross-stress tolerance (Choudhary et al, 2021), as well as the advantages of natural plant variations and new gene editing techniques (Sanchez-Bermudez et al).

The review by Aggarwal et al. updates information regarding *Setaria italica* and *S. viridis* as model plants to study broad-spectrum traits, including abiotic stress tolerance, C4 photosynthesis, biofuel, and nutritional traits. The review enumerates the trend in contemporary research of understanding climate resilience and other essential traits of Setaria, the knowledge gap in the field, and how this information could be applied to crop improvements in related millets, biofuel crops, and cereals. The review also provides a roadmap for studying other underutilized crop species using Setaria as a model.

Leaf rolling, one of the main reactions to water stress in cereals, can be visually estimated in the field (Baret et al., 2018). Moderate leaf rolling in wheat (*Triticum aestivum L*.) keeps leaves upright and maintains the relatively normal photosynthesis of plants under drought stress (Zhu et al). A candidate gene *TaMYB5-3A* that regulates leaf rolling has been identified with the aid of a genome-wide association study (GWAS) of a panel of 323 wheat accessions. Leaves from *tamyb5* mutants were more flat than wild type leaves under drought conditions. *TaMYB5-3A* was mainly expressed in leaves and down-regulated by PEG treatment. *TaMYB5* induces *TaNRL1* gene expression through direct binding to the AC cis-acting element of the target gene promoter. These results provide the theoretical basis and a gene resource for wheat crop breeding.

Besides model plants, another challenge is to identify how signaling pathways have evolved within a species to program responses with different signals and regulatory networks, giving rise to genotypes that are adapted to specific stressful conditions. Due to its excellent drought tolerance, wild barley *Hordeum spontaneum* is considered a valuable source for barley improvement. Pan et al have analysed stomatal aperture and oxidative stress markers on two barley cultivars and one wild barley accession. The authors have cloned a new *HvbZIP21* which plays a critical role in drought tolerance by regulating enzymatic ROS defenses.

There is emerging evidence that biostimulants act as plant priming agents, as demonstrated by the observed effectiveness of these formulations in promoting and sensitizing plant defenses and resistance against different environmental stresses (Nephali et al., 2020). Deciphering the priming mechanism of biostimulants is a challenging issue in agricultural science. As demonstrated by Supriya et al, melatonin (N-acetyl-5-methoxytryptamine), has a priming effect on the promotion of tolerance to drought in *Gossypium hirsutum* by modulating the antioxidant system, with increased photosynthetic parameters, water-use efficiency, and nitrogen metabolism. The higher content of the autophagosome marker, lipidated ATG8 (ATG8-PE), in melatonin-primed drought-stressed plants of sensitive cultivar L-799 indicates that autophagy plays an important role in alleviating drought stress mediated by melatonin.

Environmental changes can affect flowering and crop yield production. Thus, coffee (*Coffea arabica* L.) presents asynchronous flowering regulated by endogenous and environmental stimuli, with anthesis occurring once plants are rehydrated after a period of water deficit. López et al. have observed that the interaction of ABA-ACO-ethylene and intercellular ACC transport among coffee leaves, buds, and roots promotes an increase in ACC levels, which is most likely involved as a modulator of coffee anthesis. This study provides evidence that ACC can play an important role independently of ethylene in the anthesis process in a perennial crop. With waterlogging being a major abiotic stress during the growth cycle of maize in China, Hu et al. have observed that the application of 6-benzyladenine (6-BA) increased the yield of summer maize. At the florets differentiation stage, treatment with 6-BA increased the trans-zeatin (tZ) and salicylic acid content of ears of maize, leading to the induction of invertase activity, thus establishing sink strength. During the sexual organ formation phase, the TZ content of ear leaves, spike nodes, and ears was increased by waterlogging treatment, as compared to control conditions. Accordingly, the sugar metabolism of summer maize was also improved.

Heat stress has become a major abiotic challenge to the growth and development of various crops, leading to a reduction in their productivity. *Brassica napus*, the second largest source of vegetable oil worldwide, experiences a drastic reduction in seed yield and quality in response to heat. Kourani et al. have updated the latest research on the genetic and physiological impact of heat stress on the different developmental stages of *B. napus*, paying particular attention to the reproductive stages of floral progression, organogenesis, and post flowering. The implications of the polyploidy nature of *B. napus* and the regulatory role of alternative splicing in priming-induced heat-stress memory are presented. New insights into the dynamics of epigenetic modifications during heat stress are also discussed.

The plant cuticle is an extracellular lipid structure that covers the outer surface of land plants and protects cells from different abiotic and biotic stresses. Fukuda et al. have demonstrated that *ECERIFERUM 10/AOD2* plays a crucial role in Arabidopsis osmotolerance through very-long-chain fatty acid (VLCFA) metabolism involved in cuticular wax formation and endocytic membrane trafficking. Kajino et al., in their turn, have analysed the halophyte *Eutrema salsugineum* and identified *EsCYP78A5* as a gene that can confer acquired osmotolerance. *EsCYP78A5*, an ortholog of Arabidopsis *KLU*, encodes a cytochrome P450 monooxygenase. Transgenic Arabidopsis plants overexpressing *AtKLU* (*AtKLUox*) exhibit acquired osmotolerance and also salt-shock, oxidative, and heat-stress tolerances due to higher cutin monomer and VLCFA levels and reduced endoplasmic reticulum (ER) stress. The study by Chen et al. focus on N-acetylglucosamine-1-P uridylyltransferase (GlcNAc1pUT), encoded by *GlcNA.UTs*, which catalyzes the last step in the hexose biosynthetic pathway. The authors demonstrate that GlcNAc1pUTs are essential for protein N-glycosylation, fertility, and the response of plants to salt stress through ABA signaling pathways during seed germination and early seedling development.

Nitric oxide (NO) and its derivatives (reactive nitrogen species, RNS) are also produced in cells experiencing biotic and abiotic stress. Together, ROS and NO play key roles as biological messengers that regulate gene expression, hence activating defence responses to biotic and abiotic stresses (Romero-Puertas and Sandalio, 2016). Song et al. have analysed the contribution of S-nitrosoglutathione reductase (GSNOR), considered a critical regulator of plant stress tolerance due to its impact on protein S-nitrosylation, in the thermotolerance of *Solanum lycopersicum*. The authors conclude that GSNOR plays a role in regulating NADPH oxidase dependent (SlRBOH1) apoplastic H_2_O_2_ production in response to high temperatures, while a balanced interaction between S-nitrosothiols (SNO) and H_2_O_2_ is critical for maintaining cellular redox homeostasis and thermotolerance.

Poor soil and nutritional imbalance are major concerns worldwide which severely affect crop production and food production. Acidic soils are poor with regard to natural fertilization, thus requiring an exogenous supply of P fertilizers; however, the increase in production and transport costs, leading to an increase in the price of the final product, is a limiting factor for farmers. The study by Tarumoto et al. assesses the physiological and nutritional attributes of two sugarcane varieties in order to evaluate their efficiency against low and adequate P concentrations. Although most of the attributes analysed, including sugarcane yields, were directly influenced by P levels, RB966928 was more sensitive to low P levels and more responsive to P supply than RB867515. RB867515 appears to use the antioxidant enzyme ascorbate peroxidase to overcome P limitations, whereas RB966928 relies on increased levels of proline to increase the efficiency of photoassimilate accumulation. An understanding of these characteristics would facilitate sugarcane crop management and variety selection, especially under conditions in which P is the most limiting nutrient.

Abiotic stresses will remain a challenge to agriculture, food quality and the natural environment in the future. The use of all available phenomic, metabolomic and molecular information will facilitate the identification of gene networks involved in plant defenses against combined stress conditions. CRISPR/Cas9 is a promising tool for the precise and efficient genome editing of crop plants to combat various abiotic stresses. Thus, genetically engineered stable crops obtained by using this technique will result in increased tolerance and yields within the framework of new agronomic strategies adapted to the environment and population growth.

## Author contributions

The author confirms being the sole contributor of this work and approved it for publication.
